# Inferring Arm Movement Direction from EEG Signals Using Explainable Deep Learning

**DOI:** 10.3390/s26041235

**Published:** 2026-02-13

**Authors:** Matteo Fraternali, Elisa Magosso, Davide Borra

**Affiliations:** 1 Department of Electrical, Electronic and Information Engineering “Guglielmo Marconi” (DEI), University of Bologna, Cesena Campus, 47521 Cesena, Italy; matteo.fraternali4@unibo.it (M.F.); davide.borra2@unibo.it (D.B.); 2 Alma Mater Research Institute for Human-Centered Artificial Intelligence, University of Bologna, 40126 Bologna, Italy

**Keywords:** electroencephalography, center-out-reaching, EEG-based direction decoding, convolutional neural networks, explainable artificial intelligence

## Abstract

Decoding reaching movements from non-invasive brain signals is a key challenge for the development of naturalistic brain–computer interfaces (BCIs). While this decoding problem has been addressed via traditional machine learning, the exploitation of deep learning is still limited. Here, we evaluate a convolutional neural network (CNN) for decoding movement direction during a delayed center-out reaching task from the EEG. Signals were collected from twenty healthy participants and analyzed using EEGNet to discriminate reaching endpoints in three scenarios: fine-direction (five endpoints), coarse-direction (three endpoints), and proximity (two endpoints) classifications. To interpret the decoding process, the CNN was coupled with explanation techniques, including DeepLIFT and occlusion tests, enabling a data-driven analysis of spatio-temporal EEG features. The proposed approach achieved accuracies well above chance, with accuracies of 0.45 (five endpoints), 0.64 (three endpoints) and 0.70 (two endpoints) on average across subjects. Explainability analyses revealed that directional information is predominantly encoded during movement preparation, particularly in parietal and parietal–occipital regions, consistent with known visuomotor planning mechanisms and with EEG analysis based on event-related spectral perturbations. These results demonstrate the feasibility and interpretability of CNN-based EEG decoding for reaching movements, providing insights relevant for both neuroscience and the prospective development of non-invasive BCIs.

## 1. Introduction

Brain–computer interfaces (BCIs) have emerged as a technology enabling direct communication between neural activity and external devices, offering promising solutions for the restoration or augmentation of motor and cognitive functions [1,2,3]. Recent advances in neural decoding aim to improve the precision, speed, and usability of BCIs, particularly in applications such as neuroprosthetic controls and assistive systems for individuals with severe motor impairments. To achieve more naturalistic motor BCIs, reaching movements are extensively studied, as they underpin everyday interactions between users and their environment and serve as a foundation for more complex actions, such as reach-to-grasp movements.

Recent studies have attempted to reconstruct kinematic parameters, such as hand trajectory and reaching endpoints, from invasive neural signals (via intracortical microelectrode arrays, electrocorticography, or single-unit recordings) [4,5,6,7], achieving state-of-the-art performance for motor reaching decoding. Unfortunately, the clinical application of invasive motor BCIs remains constrained by surgical risks, biocompatibility issues, and ethical considerations. Indeed, invasive studies are predominantly conducted in non-human primates or pathological populations, thus providing limited insights into the neural organization of motor control in healthy individuals.

The investigation of motor reaching decoding in healthy participants constitutes a necessary foundation for understanding the normal architecture of motor control and for guiding the development of BCI models [8]. In this context, non-invasive neuroimaging techniques provide an invaluable approach. Electroencephalography (EEG) remains the most accessible and widely used modality for BCI research due to its high temporal resolution and portability, and direct measurement of neuronal electrical activity. Although EEG suffers from reduced spatial resolution compared to invasive methods, it captures meaningful oscillatory patterns—such as alpha-band and beta-band event-related desynchronizations and synchronizations (ERD/S) [9]—and event-related potentials—such as motor-related cortical potentials [10]—that can be exploited to infer motor planning and execution.

Advances in the field of EEG-based motor decoding involve the use of machine learning approaches [11]. In these approaches, handcrafted features are first extracted (e.g., alpha-band power of pre-selected EEG channels) and then classified into the target motor states by a learning system (e.g., support vector machines or linear discriminant analysis). The simplest EEG-based reaching decoding scenario targets the classification of highly separated motor states—for example, leftmost vs. rightmost direction of center-out reaching [12,13]. Finer direction decoding of center-out-reaching is also addressed, by classifying from four distinct endpoints (left, right, up, down) [14,15,16,17] to eight endpoints equally spaced on a circumference [14]. Crucially, all these studies considered standard machine-learning decoding pipelines, by selecting a priori EEG features to be classified with a linear or non-linear learning system. Because they rely on handcrafted feature extraction, often guided by a priori assumptions, these approaches fail to fully exploit the information contained in EEG data. In particular, handcrafted features typically rely on predefined temporal windows, frequency bands, and spatial channel selections. However, EEG signals during complex reaching movements likely contain task-relevant information distributed dynamically across time, frequency, and scalp locations. Under these conditions, fixed feature representations may fail to adequately represent the richness of the neural patterns associated with the task. As a result, they may not capture the complete organization of motor control and may overlook discriminative neural signatures. Consequently, the use of these approaches does not allow the data-driven (i.e., guided from data) and end-to-end (i.e., directly mapping all the EEG signals to the motor states under analysis) investigation of directional encoding in EEG signals during center-out reaching.

In the last decade, research has been directed towards the design of models capable of automatically extracting from EEG data the most informative neural features for general EEG decoding, and jointly solving the classification problem. Among these promising models, convolutional neural networks (CNNs) have emerged as particularly effective [18]. EEGNet [19] and its variants [20,21,22,23] represent the most used CNN architecture for general-purpose EEG decoding, providing the best trade-off between model size (i.e., number of trainable parameters), training time, and decoding performance [24], also reaching state-of-the-art performance in various international EEG decoding competitions [20,21]. CNNs can be coupled with explanation techniques (e.g., DeepLIFT [25] and occlusion tests [26]) to increase the interpretability of the decision-making process [27], for example by highlighting the most influential time samples and brain regions contributing to the network’s output.

Despite these premises of deep learning approaches, the directional information in center-out reaching tasks has been primarily decoded via machine learning approaches, and the potential of CNNs for reaching decoding still needs to be investigated.

In this work, we aim at filling this gap, by applying a CNN-based decoder to discriminate directions of center-out reaching in different setup scenarios (from two to five reaching endpoints). EEG signals were recorded from twenty healthy participants in a delayed center-out reaching task, and a CNN based on EEGNet was employed to decode the reaching endpoints. The CNN was coupled with explanation techniques (DeepLIFT and occlusion tests) to automatically reveal how directional reaching information is encoded in EEG signals in space and time. This way, without injecting a priori information, the most relevant spatio-temporal EEG features of center-out reaching can be revealed, in a data-driven and end-to-end way. The so-obtained results are also discussed in light of traditional spectral analysis of the EEG data (event-related spectral perturbation). Therefore, this work presents a pipeline that combines end-to-end deep learning with explainable techniques and established spectral analyses, aiming for a neurophysiological comprehension of the network’s decisions, and thereby contributing to the field of trustworthy and interpretable AI in EEG-based decoding.

With this work, we expect to contribute to the investigation of fine EEG-based motor decoding for prospective naturalistic non-invasive BCIs, and to advance our knowledge about the motor encoding of center-out reaching.

## 2. Materials and Methods

### 2.1. Participants

In this study we used the data recorded by Borra et al. [28]. Twenty healthy volunteers (11 males, 9 females; age = 21.9 ± 2.3 years, mean ± standard deviation) took part in the study. All participants were right-handed and had normal or corrected-to-normal vision. The experimental protocol was approved by the Bioethics Committee of the University of Bologna (protocol code 61243; approval date: 15 March 2021), and written informed consent was obtained from each participant before the start of the experiment. All data were collected, processed, and reported in an anonymized form.

### 2.2. Experimental Protocol

**Figure 1:**
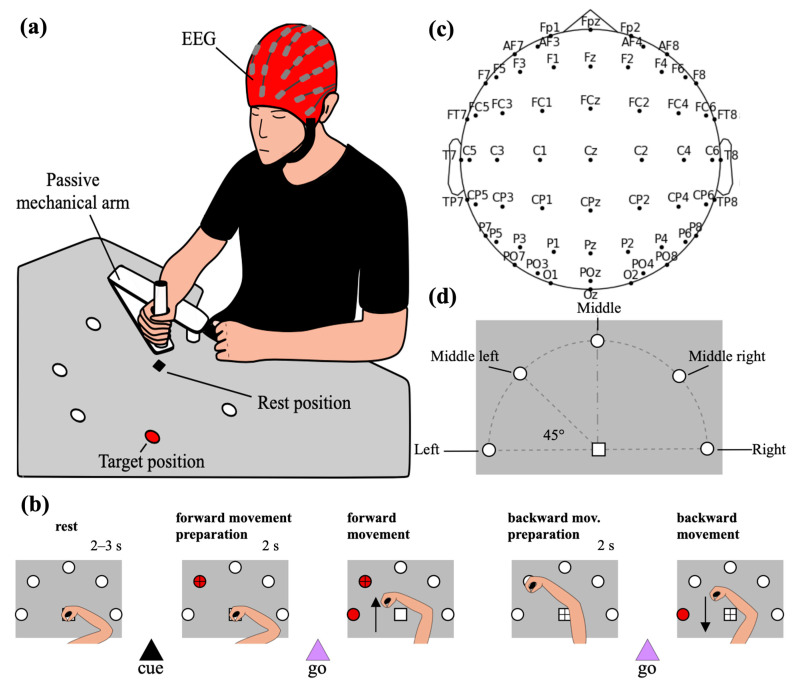
Delayed center-out-reaching task. (**a**) Schematics of the recording set-up. (**b**) Trial timeline. Each trial started with the participants having the arm in the rest position (indicated by a white square in the center of the semi circumference) and fixating on the rest position. The white circles represent the five possible positions to be reached by the forward movement. Each position consisted of a red LED, normally turned off (white circles represent the red LEDs when turned off). The participants waited in the rest position for an interval with a random duration between 2 and 3 s (first panel on the left). Then, the cue signal was presented, consisting of the red LED turning on at the target position (dark red circle with a cross in the second panel on the left), and the participants shifted eye fixation to the target LED, preparing for the movement. After a 2 s preparation phase, the adjacent LED illuminated (dark red circle, adjacent to the target, in the central panel), serving as the go signal for executing the forward movement. When the target position was reached, all LEDs turned off (represented by all circles returning white in the figure) and the participants shifted the fixation to the rest position (which became the position to be reached by the backward movement), waiting for another go signal before performing the backward movement (second panel from the right). After 2 s, the same go LED as for the forward movement was turned on again (dark red circle in the last panel on the right), serving as the go signal for executing the backward movement. The arrow in the ‘forward movement’ and ‘backward movement’ panels has only illustrative purpose and serves to distinguish the direction of the movement. (**c**) EEG electrode positions based on the 10–10 reference system. (**d**) Names of the target positions (reaching endpoints).

Participants performed a delayed center-out reaching task, involving reaching movements toward one of five target positions with the dominant arm (right arm for all participants). The positions were arranged horizontally on a table, along a semicircular arc (radius: 40 cm) positioned in front of the participant. Targets were spaced evenly at 45° intervals along the semicircle, with the center of the semicircular arc corresponding to the participant’s starting hand position (Figure 1a,d). Participants were seated comfortably in front of the table, performing movements on the table plane. Each target position was equipped with a red LED, which was turned on to indicate the position the participant had to reach. The selection of the target position was performed using a DAQ NI USB-6008 board (National Instruments Corp., Austin, TX, USA) controlled via MATLAB R2021b (The Mathworks Inc., Natick, MA, USA). The reaching movement was supported by a custom-built passive mechanical arm designed to have low friction, to reduce fatigue and ensure a smooth movement.

The experimental session consisted of six recording blocks, each comprising 50 trials. A short break was inserted between blocks, depending on the participants’ self-reported fatigue. Each block included 10 repetitions of the reaching movement toward each of the five target positions. Within each block, the sequence of target positions to reach was randomized. In total, each participant completed 300 trials, with 60 trials per target position. A schematic representation of the single trial timeline is shown in Figure 1b. Each trial began with the participant having the hand in the resting position (center of the semicircular arc), while maintaining the fixation on this point. After a random delay of 2–3 s from the beginning of the trial, the target LED turned on (cue signal), and the participants were instructed to shift their eye fixation from the resting position to the target. After 2 s from the cue signal, the start signal for the forward movement was provided by the illumination of an adjacent LED (go signal), and the participants were required to perform the forward center-out reaching movement. Once the target was reached, the two LEDs turned off and the participants returned their gaze to the resting position, waiting for another go signal before performing the backward movement. After 2 s, the go signal for the backward movement was provided (turning on the same LED used as the go signal for the forward movement), signaling the participant to initiate the backward movement and return to the resting position.

A 60-channel EEG system (g.HIamp Research amplifier, g.tec Medical Engineering GmbH, Schiedlberg, Austria) was used to record the EEG signals. At the beginning of the experiment, the task was explained to the participants and the EEG cap with 60 active electrodes (g.SCARABEO electrodes; g.tec Medical Engineering GmbH, Schiedlberg, Austria) was mounted according to the international 10–10 system (Figure 1c). The reference electrode was placed on the earlobe, and the ground electrode for noise reduction was placed at AFz. Conductive gel was applied to maintain electrode impedance below 50 kΩ. EEG signals were sampled at 512 Hz. A notch filter (stopband: 42–58 Hz) was applied during recording.

### 2.3. EEG Data Analysis

In this study, the analysis was focused on classifying the forward movements from the recorded scalp EEG signals using a deep learning approach. We examined both the preparation phase and the execution phase of the forward movements. This was important to discriminate the EEG representation of the movement in the planning phase, which encompasses visual processing of the target location and its translation into motor commands from the execution phase, which involves the commitment to movement and motor control processes. Although the focus was on the forward movements, a complimentary control analysis was performed on the backward movements (preparation phase only) to support result interpretation.

All the following steps of the EEG analysis were implemented offline using Python (Python 3.12.12, MNE version 1.10.2 [29], PyWavelets version 1.9.0 [30], PyTorch version 2.9.0 [31], Captum version 3.10.0 [32]).

#### 2.3.1. EEG Preprocessing

Data of each participant were preprocessed following the pipeline described in Borra et al. [28]. Specifically, the raw 60-channel EEG signals of each block first underwent linear detrending to remove slow drifts, followed by band-pass filtering (1–60 Hz) and notch filtering (50 Hz) to attenuate residual line noise and retain the relevant frequency components. Bad channels were identified within each block using the random sample consensus algorithm [33]. Then, the block signals were concatenated and the channels that produced a bad result in at least one block were removed. Independent component analysis (ICA), implemented via the extended Infomax algorithm [34], was applied to the concatenated signals (without bad channels) and artifact removal was then performed. In the inspection of artifact-related ICs, particular attention was given to identification and removal of components associated with ocular activity, in particular ocular movements, given the potential risk of directional information leakage due to shifting eye fixation at the cue presentation. In addition, all ICA-preprocessed EEG data were visually inspected to ensure the absence of residual ocular movement artifacts, after the elimination of artifact ICs. Finally, the signals of previously removed bad channels were reconstructed from the artifact-free signals using spherical spline interpolation.

The continuous preprocessed EEG signals were then segmented into epochs to extract the portions of the signals corresponding to the preparation and execution of the forward movements. Epochs of 5 s, ranging from 3 s before to 2 s after the go signal of the forward movement were extracted (300 EEG epochs in total, one per trial). This time interval was denoted as −3 s to +2 s, where 0 s corresponded to the go signal of the forward movement. Since the cue signal preceded the go signal by a fixed interval of 2 s, each epoch included 1 s of baseline (from −3 s to the cue signal, i.e., from −3 s to −2 s), the 2 s interval of preparation (the interval from −2 s to 0 s, from the cue signal to the go signal) and a 2 s interval after the go signal (from 0 s to +2 s) during which the forward movement was executed. Baseline correction was applied on a trial-by-trial basis by subtracting the mean value of the 1 s baseline (from −3 s to −2 s) for each channel.

The previous epoching stage was designed to analyze the forward (center-out) reaching movement which was the main focus of our study. However, we performed an additional epoching stage to the same continuous preprocessed EEG signals to extract portions of the signals corresponding to the backward movement. Specifically, epochs of 2 s were extracted starting 2 s before the go signal of the backward movement and ending at the go signal itself (300 epochs in total, one per trial). Therefore, 2 s epochs containing the preparation phase of the backward movement were obtained. This time interval was denoted as −2 s to 0 s; here 0 s corresponded to the go signal of the backward movement. Baseline correction was applied on a trial-by-trial basis by subtracting the mean value of the 1 s baseline (for each backward movement epoch, the same baseline of the corresponding forward movement epoch was used). As described in the Results Section (Section 3.3), the backward movement preparation epochs were used to perform a control analysis aimed at supporting the interpretation of the CNN-based results obtained during the movement preparation phase.

Finally, the epoched data were re-referenced to the common average reference and downsampled to 128 Hz to reduce the computational cost of the following analysis steps. After careful artifact removal using ICA in the previous preprocessing steps, no additional trial rejection criteria were applied, since visual inspection at the end of the preprocessing steps did not reveal residual artifacts that would justify excluding trials. This also avoided the use of arbitrary thresholds for trial rejection and preserved full balance (same number of epochs) across the five targets (see also Section 2.3.3 for class balancing in the addressed decoding problems).

The following (Section 2.3.2, Section 2.3.3 and Section 2.3.4) describe the analyses relative to the forward movement (spectral analysis in Section 2.3.2, CNN-based decoding and analysis in Section 2.3.3 and Section 2.3.4). The last section (Section 2.3.5) describes the additional CNN-based decoding and analysis applied to the backward movement epochs.

#### 2.3.2. Scalp Event-Related Spectral Perturbation

Initially, we performed a preliminary analysis aimed at depicting the temporal and spatial dynamics of the alpha-band (8–13 Hz) and beta-band (13–30 Hz) changes during the forward movement preparation and execution phase. These two bands were considered as they are primarily implicated in visuomotor processes [9]. Results of this analysis served to better contextualize the outcomes of the deep learning-based approach and to relate these outcomes with the observed spectral perturbations.

For each subject, trial, and EEG channel, the event-related spectral perturbation (ERSP) was computed to quantify time-resolved changes in oscillatory brain activity associated with task events. To this aim, continuous wavelet transform was applied to each EEG signal using the complex Morlet wavelet as the mother function (normalized bandwidth: 1.5; normalized center frequency: 1.0). Complex wavelet coefficients were squared to obtain a time-frequency representation of signal power. For each subject and channel, the power values were then averaged across the forward movement epochs (−3 s to +2 s around the go signal for the forward movement) separately for each target position to obtain position-specific spectral modulations. A normalization was applied using the baseline interval (i.e., the 1 s interval before the cue signal, from −3 s to −2 s). Specifically, the baseline power for each frequency was computed as the mean within this interval. The ERSP was then calculated as the difference between the power at each time-frequency point and the average baseline power at the same frequency, divided by this same average baseline power, producing a normalized measure of spectral modulation ($ERSP\left(t,f\right)\;=\;\frac{Power\left(t,f\right)\;-\;Baseline\left(f\right)}{Baseline\left(f\right)}$).

To examine band-specific dynamics, ERSP values were extracted for the alpha and beta bands by averaging across the respective frequency ranges. These data were then used to generate scalp maps showing the time evolution of alpha-band and beta-band ERSP during forward movement preparation and execution separately for each of the five target positions. Temporal evolution was discretized into 0.5 s non-overlapped windows (10 windows in the interval −3 s to +2 s), with the band-specific ERSP power averaged within each window.

Finally, statistical tests were applied to assess for statistically significant differences in alpha-band and beta-band ERSP among the different target positions. For each considered time window and each EEG band, a pairwise two-sided permutation *t*-test [35] was conducted separately between each pair of target positions, channel by channel. *p*-values were corrected via false discovery rate Benjamini–Hochberg (FDR-BH) procedure [36] for multiple tests (60 multiple tests, for each time window, each pair of target positions and each band).

#### 2.3.3. Deep Learning-Based Classification

**Figure 2:**
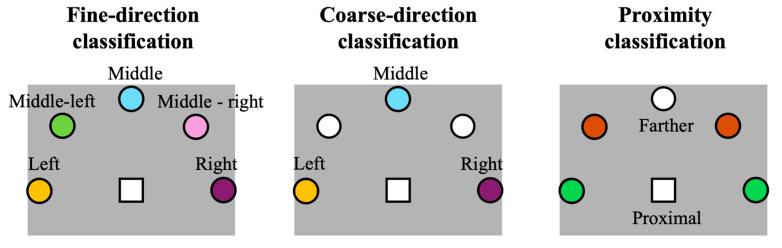
Classification problems addressed for the forward movement. Each panel displays the resting position of the hand (white square) and positions of the targets (circles). In each panel, targets that are colored (i.e., not white) are included in the classification, and different colors are used to indicate separate classes. Fine-direction classification: all five target positions were considered as separate targets (i.e., all forward movement epochs were considered, and each epoch was classified as belonging to one of the five classes). Coarse-direction classification: only left, middle and right target positions were considered (i.e., forward movement epochs corresponding to these three target positions were considered, and each epoch was classified as belonging to one of the three classes). Proximity classification: the left and right target positions were considered together as forming the class proximal to the frontal plane of the participant, while the middle left and middle right target positions were considered together as forming the class farther from the frontal plane of the participant (i.e., forward movement epochs corresponding to these target positions were considered, and each epoch was classified as belonging to one of the two classes). The middle target position was excluded to avoid class imbalance, as it would contain half the number of epochs compared to the other two classes (see also the text).

In this study, we selected EEGNet [19] to classify between the target positions of the forward movement. Three different classification problems were considered to address distinct decoding problems, as depicted in Figure 2. A first classification problem (fine-direction classification) considered all five target positions as separate output classes. This problem explored fine EEG decoding of forward movement direction. The classes were balanced based on the experimental paradigm and preprocessing steps. The second classification problem (coarse direction classification) classified targets into three categories, left, middle, and right, by using the forward movement epochs corresponding to the 90° spaced targets (discarding the intermediate positions middle left and middle right). Here, a coarser direction decoding of forward movements was addressed. In this case too, the classes were balanced. Finally, a third classification problem addressed the classification of proximal vs. farther targets (proximity classification). In this case, the target positions left and right were combined to represent the positions closest to the participant’s frontal plane, while the target positions middle left and middle right were combined to represent positions farther away from the frontal plane. This last problem was designed to assess movement decoding as a function of proximity relative to the participant’s frontal plane, disregarding the hemispace (left or right). The two considered classes were balanced. The middle target was excluded from this classification problem to avoid unbalanced classes, since the class corresponding to the middle target would have half the epochs of the other two classes, which collapsed together epochs associated to two targets.

In each classification problem, EEGNet received as input the signals $X_{i}$ of a trial ($X_{i}\in R^{C\times T}$, where C = 60 is the number of channels, T = 640 is the number of time samples), and provided as output the conditional probabilities that the trial belonged to each of the output classes (e.g., in case of the 3-class classification problem, the network provided as output $p\left(\left.l_{o}\right|X_{i}\right)$, ${\forall l}_{o}\in L=\left\{left,middle,right\right\}$). The architectural parameters of EEGNet are summarized in Table 1.

In each classification problem, we adopted a within-subject training strategy, using a 5-fold cross validation scheme on each participant-specific EEG dataset. In each fold, the 20% of the examples of the training set was used as validation set to arrest the learning at the training epoch with the highest validation accuracy. The categorical cross-entropy was used as a loss function. Trainable parameters were optimized using the Adam optimizer, with mini-batch training [37] (up to 250 epochs, learning rate of 1 × 10^−4^, mini-batch size of 64). For model evaluation, we employed multiple metrics to obtain a comprehensive assessment of classification performance. The following metrics were used: confusion matrix, accuracy, F1-score, and the AUC (area under the receiver operating characteristic curve). For each participant, these metrics were computed on the test set examples of each cross-validation fold and then averaged across folds. Therefore, performance metrics are provided for unseen examples (belonging to the held-out test set).

Using the available hardware (Google Colab T4 GPU), the training of the network took approximately 10 min per participant for fine direction classification, totaling around 3.3 h for all 20 participants. For coarse direction classification and proximity classification, the training time was 8 min × 20 participants = 160 min (2.7 h) and 5 min × 20 participants = 100 min (1.7 h), respectively.

#### 2.3.4. Explanation Techniques

To better understand the internal decision mechanisms of the trained deep learning model, we applied a post hoc explanation technique. Specifically, this was used to identify the input samples in the time and space domains that mostly drove the network decision towards the correct forward movement class. For each trained neural network (i.e., for each participant and each cross-validation fold), the model decisions were explained using the Deep Learning Important Features (DeepLIFT) algorithm [25], while the network processed the test set examples $X_{i}$ as input (i.e., $\forall i|X_{i}\in test\;set$). After the forward propagation of each input example $X_{i}$, DeepLIFT backpropagates the output prediction to a target layer (e.g., the input layer), providing a relevance representation map with the same shape of the target layer, quantifying the positive or negative contribution to the output prediction. DeepLIFT computes these relevance values by measuring the change in the network output relative to a reference output, in response to the change in the input from a reference input. In this study, DeepLIFT was selected as the explanation technique because a recent benchmark highlighted it as the most effective approach for EEG analysis [38]. A complete mathematical formulation of the DeepLift algorithm can be found in [25].

We derived DeepLIFT relevance representations associated to the output neuron of the correct class with respect to the input layer. A zero-valued reference input was used, corresponding to the default configuration of DeepLIFT. For each trained network (i.e., for each participant and each cross-validation fold), an input relevance map (with the same size of the input EEG) was obtained for each input test example, and the absolute values were computed. The absolute values were used, since we were interested in highlighting input samples that strongly influenced the correct output class, either positively or negatively. The maps were averaged across the test trials, and then across folds, resulting in a spatio-temporal relevance map per subject (spatio-temporal relevance). This map can be visualized as a heatmap, highlighting both temporal and spatial relevance patterns. To emphasize these two aspects separately, two additional representations were computed for each subject by preserving only the temporal dimension (i.e., by averaging the spatio-temporal relevance across channels—temporal relevance) or the spatial dimension (i.e., by averaging the spatio-temporal relevance across time samples within a selected time window—spatial relevance). A pairwise two-sided permutation *t*-test (corrected using the FDR-BH method) was applied to determine significant deviations of the time samples from the baseline (estimated in the −3 to −2 s interval).

Besides DeepLIFT, we applied an additional explanation technique consisting of post hoc occlusion [26] to disentangle the specific contributions of the preparation and execution phase of the forward movement to network performance. To this end, test trials were fed to the trained models while selectively replacing the EEG signals with zeros either during the preparation interval of the forward movement (from −2 s to 0 s, before the go signal) or during the execution interval (from 0 s to +2 s, after the go signal). Model performance under each occlusion condition was then compared to that obtained using the non-occluded test inputs. Specifically, differences were computed between the confusion matrix derived from the complete test inputs and those derived from the occluded inputs either in the preparation or execution interval, thereby assessing the relative contribution of each forward movement phase to the model’s performance. Statistical analysis using paired *t*-test and FDR-BH correction was performed to identify differences between the occluded and non-occluded test set.

#### 2.3.5. Complementary Analysis Applied to the Backward Movement Preparation Phase

The following analyses were applied to the backward movement preparation epochs. First, we applied the EEGNet to decode backward movement preparation epochs, addressing two classification problems: the coarse direction classification (3-class problem) and the proximity classification (2-class problem). In this case, the network discriminated between the backward movement preparation from the different reached positions to the rest position. The same methodology used for the forward movement decoding was applied here for the backward movement decoding (see Section 2.3.3). Second, we applied DeepLIFT to identify the temporal and spatial samples of the backward movement preparation epochs that contributed most to the discrimination (in each of the two classification problems), using the same methodology used for the forward movement (see Section 2.3.4).

To compare the results achieved in decoding the forward movement preparation phase and the backward movement preparation phase, a paired *t*-test was applied comparing the decoding accuracy between the two conditions, separately in each classification problem. Specifically, we considered the accuracy values obtained for the forward movement epochs while occluding the motor execution phase. In this way, the accuracy was computed considering test examples, including only the 2 s length portion of the epoch corresponding to the preparation phase, both for the forward and backward movements.

## 3. Results

Section 3.1 and Section 3.2 describe results relative to the forward movement epochs, while Section 3.3 describes the results of the complementary analysis applied to the backward movement preparation epochs.

### 3.1. Scalp Event-Related Spectral Perturbation

**Figure 3:**
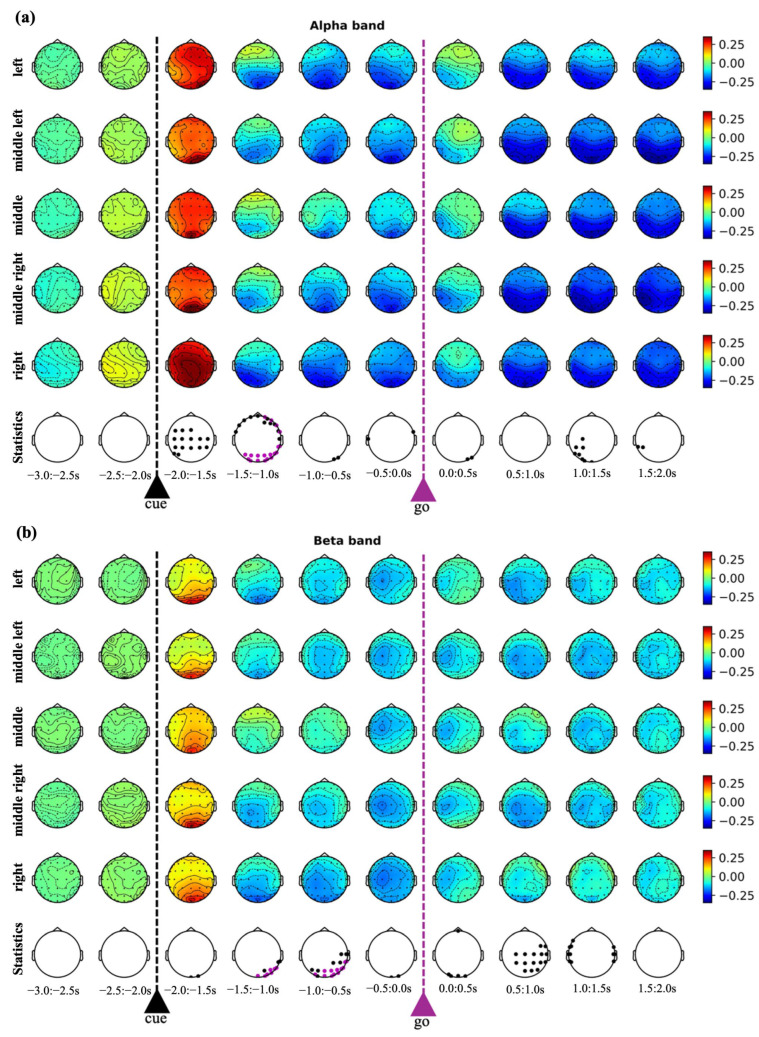
Grand average scalp maps of event-related spectral perturbation (ERSP). Panel (**a**) shows the ERSP in the alpha band. Panel (**b**) shows the ERSP in the beta band. In each panel, rows from 1 to 5 report the scalp topographies of the ERSP associated with the different target positions (left, middle left, middle, middle right, right) represented in 0.5 s time windows across the epoch. The represented ERSP were unitless since we adopted a normalization relative to the baseline (see text for details). The maps show an evident desynchronization after the cue signal and after the go signal, more posteriorly than the alpha-band and more centrally located than the beta-bands. Additionally, a clear left-lateralization pattern emerges in the time window immediately after the go signal (0, 0.5 s). The last row of white maps in each panel (‘Statistics’ row) shows the results of the statistical comparisons. For each time window a pairwise permutation *t*-test was conducted for each pair of target positions, to identify the electrode locations at which the ERSP was statistically different (*p* < 0.05). The electrode locations producing a significant result in at least one comparison were displayed as black dots inside the white scalp maps. Violet dots mark electrodes that remained statistically significant even after FDR-BH correction.

Figure 3 shows the ERSPs for the alpha (Figure 3a) and beta (Figure 3b) frequency bands.

In the alpha band, a clear ERS was observed immediately after the cue signal (−2 s to −1.5 s), involving the whole scalp but peaking at parietal and occipital electrodes. This response likely reflected alpha-band components embedded into the event-related potential elicited by the cue indicating the target position. Subsequently (−1.5 s to 0 s), alpha ERD developed, predominantly over posterior and central regions. The go signal did not evoke an initial ERS but was instead immediately followed by an ERD, indicating a qualitatively different neural processing of the two types of visual stimuli. Early after the go signal (0 s to 0.5 s), the ERD was mainly localized over the left parieto-occipital and central scalp sites, consistent with the triggering of right-hand movements. As the movement unfolded, the ERD increased in magnitude and broadened spatially throughout the entire execution phase, suggesting a continuous involvement of sensorimotor regions during task performance. From the statistical analysis on the alpha ERSP, the ERD observed during movement preparation was significantly different between reaching endpoints in the −1.5 s to −1.0 s window, involving parietal/occipital electrodes (see violet dots in Figure 3, marking comparisons that survived after multiple-tests correction).

In the beta band (13–30 Hz), the temporal evolution of the ERSPs mirrored that of the alpha band, though with a generally lower amplitude. Upon cue presentation (−2 s to −1.5 s), beta-band ERS occurred, mainly concentrated over posterior regions. This was followed (−1.5 s to 0 s) by beta ERD similarly as to the alpha-band, but with the beta ERD having a more central spatial localization and a left-lateralization tendency, especially at the end of the preparation phase (−0.5 s to 0 s). Beta ERD with a central left-lateralized pattern was also evident early after the go signal (0 s to 0.5 s reaction period). Then, as the movement was initiated (approximately from 0.5 s onward), beta ERD tended to spread, still remaining more pronounced over central electrodes. Similar to the alpha band, from the statistical analysis on the beta ERSP, the ERD observed during movement preparation significantly differed between reaching endpoints in the −1.5 s to −1.0 s and −1.0 s to −0.5 s windows, involving parietal/occipital electrodes (see violet dots in Figure 3).

### 3.2. Deep Learning Classification and Analysis

**Figure 4:**
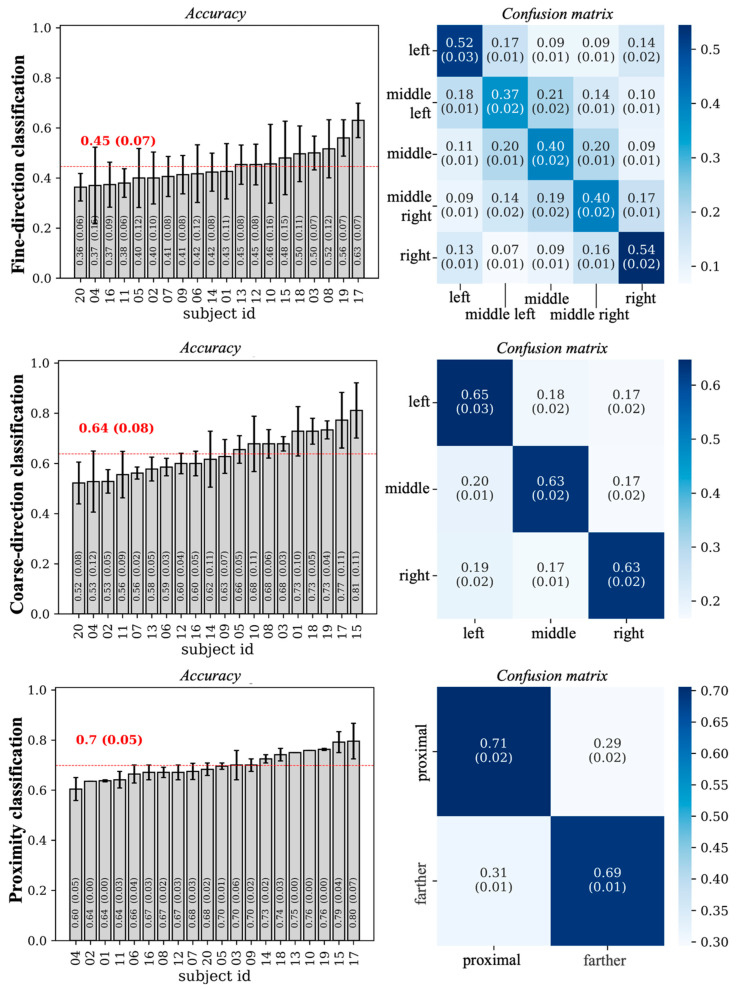
Neural network performance metrics: accuracy and confusion matrix. The accuracy and confusion matrix scored in each classification problem are reported. The classes used in each classification problem are depicted in Figure 2 and explained in Section 2.3.3. In the left panels, accuracy bars display the mean (bar height) and standard deviation (black segment) across the 5 folds for each subject. The red dashed line represents the mean accuracy across all subjects (standard deviation in parentheses). Note that in each barplot, single subject accuracies were not sorted according to the subject ID, but in ascending order. In the right panels, each confusion matrix displays the proportion of test examples from each true class that were predicted as belonging to each possible class (true classes along the rows, predicted classes along the columns). In each cell of the matrix, the mean (standard deviation) across subjects is reported.

Figure 4 presents the single-subject accuracy and the confusion matrix for each of the three classification problems. Accuracy values were sorted in ascending order by subject. In all three cases, the metrics achieved values above the chance level (0.5 for the proximity classification task, 0.33 for the coarse direction task, and 0.2 for the fine direction classification). The fine direction classification problem is particularly challenging; therefore, modest performance metrics are to be expected. The confusion matrix provides a more detailed view of model performance by reporting the proportion of examples of one true class predicted as belonging to each possible class. As expected, the highest values appeared along the main diagonal, while larger errors occurred in the misclassification of neighboring positions. For completeness, additional performance metrics, including F1-score and AUC, are reported in Table 2.

**Figure 5:**
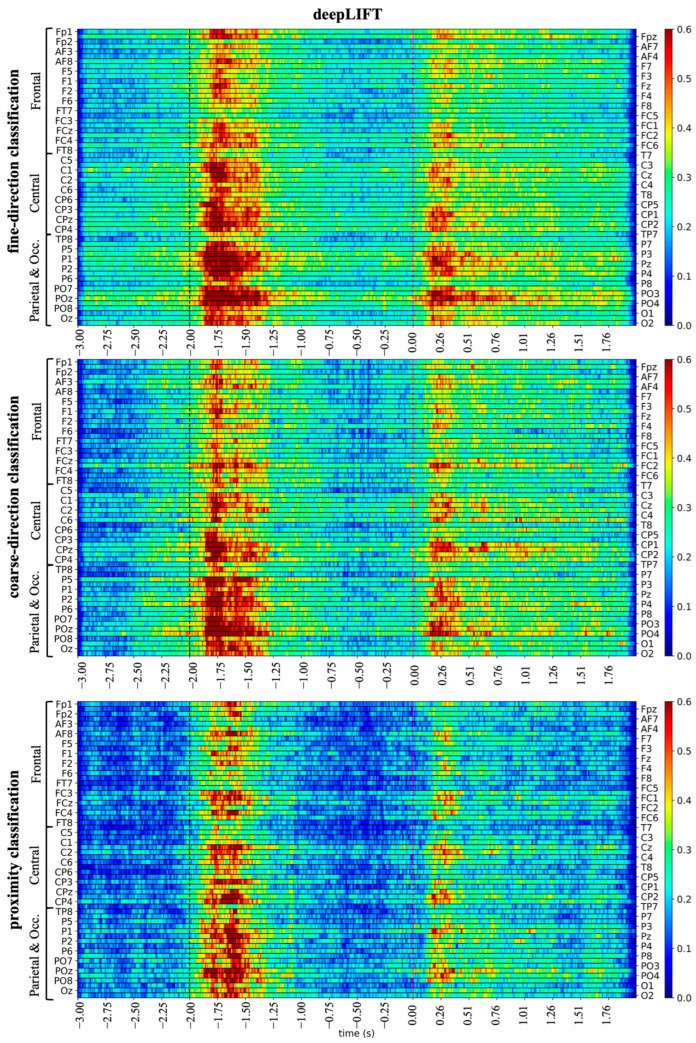
Explaining network decision: spatio-temporal relevance. Spatio-temporal relevance representations are displayed as heatmaps (channels along rows, time samples along columns), separately for the fine-direction, coarse-direction and proximity classifications. In each panel, each horizontal line displays the time relevance for a specific channel (60 channels per panel, overall). To show the labels for all the 60 channels and ensure readability, half of the labels were displayed along the left *y*-axis and the other half along the right *y*-axis, by alternating one label on the left and one on the right. The two vertical dotted lines show the cue signal (black line) and go signal (purple line). Channels are grouped into 3 regions: Frontal, Central and parietal–occipital (Parietal & Occ.).

**Figure 6:**
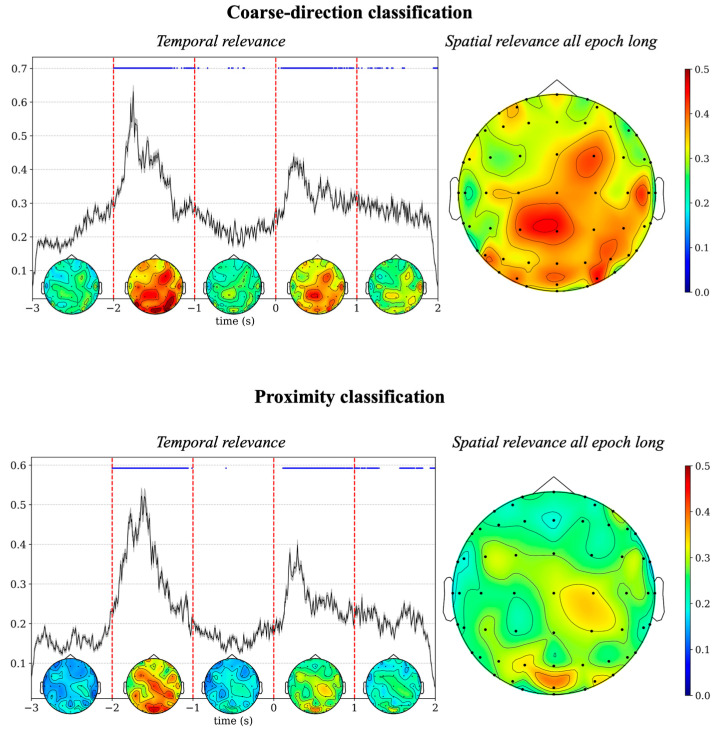
Explaining network decision: temporal and spatial relevance. Relevance representations are displayed in the temporal and spatial domains for the coarse-direction classification and the proximity classification. In the left panels, the plots display the temporal relevance aggregated across subjects (black line: mean value; shaded area: standard error of the mean). The temporal relevance in each time sample within the interval −2 s to +2 s was tested for significant difference compared with the baseline (average relevance in the interval −3 s to −2 s, see Section 2.3.4): significant time points are indicated by blue dots at the top of the figure, which may appear as a continuous line when adjacent samples are significant. Within 1 s intervals (5 in total), delimited by dashed red lines, the spatial topology of the relevance in distinct time windows is reported, by computing the average of the spatio-temporal relevance in time. In the right panels, the spatial relevance is displayed, aggregated across subjects (mean value).

**Figure 7:**
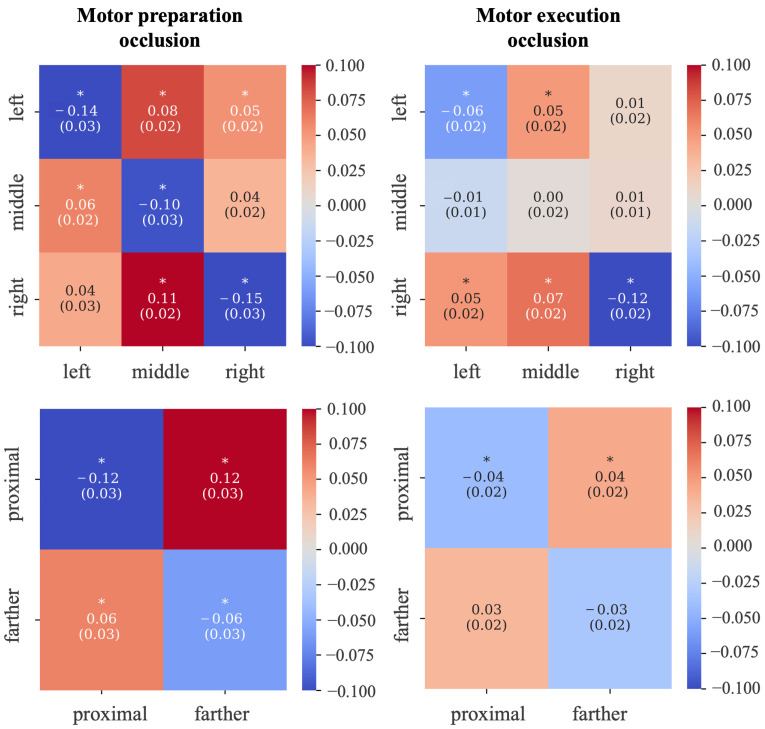
Explaining network decision: occlusion tests. The left panels and right panels respectively show the performance results while occluding the motor execution phase and preparation phase separately for the coarse-direction decoding (**first row**) and proximity decoding (**second row**) problems. Each confusion matrix displays the differences between the performance scored by the model on the occluded examples and the same by the model on the non-occluded examples (see Figure 4). Negative values indicate a decrease in accuracy, whereas positive values indicate an improvement. Asterisks indicate significant differences (*p* < 0.05 after FDR-BH correction).

The post hoc explainability results based on DeepLIFT are summarized in Figure 5 and Figure 6, while those based on occlusion tests are summarized in Figure 7. Specifically, Figure 5 illustrates the spatio-temporal relevance maps separately for each classification problem. These representations, although qualitative (i.e., without any statistical thresholding), exhibited peaks of relevance a few milliseconds after the cue and go signals. Since the 5-class classification problem yielded relatively limited accuracy, the next visualizations (Figure 6 and Figure 7) are provided for the coarse-direction and proximity classifications for brevity, where the models achieved more reliable performance. Temporal relevance maps and spatial relevance maps are visualized in Figure 6, which were obtained by averaging the corresponding map in Figure 5, either in the time domain or spatial domain (see details in Section 2.3.4). Temporal relevance maps indicate that the most relevant input features occur primarily early after the cue signal (−2 s to −1 s) and early after the go signal (0 s to 1 s), with statistically significant differences (corrected *p* < 0.05). Spatial relevance maps reveal that posterior regions (centro-parietal/parietal/parietal–occipital) contribute strongly to model predictions.

Overall, from spatio-temporal relevance maps (Figure 5) and individual domain relevance maps (spatial and temporal, Figure 6), the preparatory phase appeared more relevant than the execution phase. The specific contributions of the preparatory phase and motor execution phase (i.e., distinct time intervals) were also evaluated in a post hoc occlusion procedure, by comparing the performance obtained using input examples in which each phase was occluded against the performance obtained using the non-occluded examples. Figure 7 reports the differences between the confusion matrix obtained using examples either with the motor preparation phase occluded (left panels) or with the execution phase occluded (right panels) and the confusion matrix obtained with the non-occluded examples.

Results show that the preparatory phase plays a significant role in decoding performance. Maintaining this interval while removing the execution phase led to a relatively small decrease in overall accuracy, typically of only a few percentage points, indicating that the network greatly relied on predictive information available during the preparation period. In contrast, occluding the preparatory phase and maintaining the motor execution phase resulted in a more substantial reduction in accuracy across both classification tasks, suggesting that execution-related signals provided complementary information for correct classification, but the network relied more on the motor preparation phase. Overall, occluding the preparation phase reduced accuracy by an average of 13% and 9% in the coarse-direction classification problem and proximity problem, compared to 6% and 3.5% respectively in case of execution phase occlusion.

### 3.3. Complementary Analysis for the Backward Movement Preparation Epochs

An additional analysis was applied to the backward movement preparation phase to clarify the possible contribution of visual stimuli processing in the results. Indeed, the decoding of the forward movement preparation could be influenced by the visual spatial encoding of cue appearance, which consisted of the LED at the target position turning on. Therefore, this introduced the possibility that during movement preparation (especially in the early preparation phase, −2 s to −1 s), the CNN decoding could be mainly based on the visual evoked response, rather than on motor intention itself. To directly test this possibility, we performed additional control analyses on the backward movement preparation phase. Indeed, during this phase, no differences in visual stimuli across classes occurred, while the motor vector differed depending on the previously reached target.

**Figure 8:**
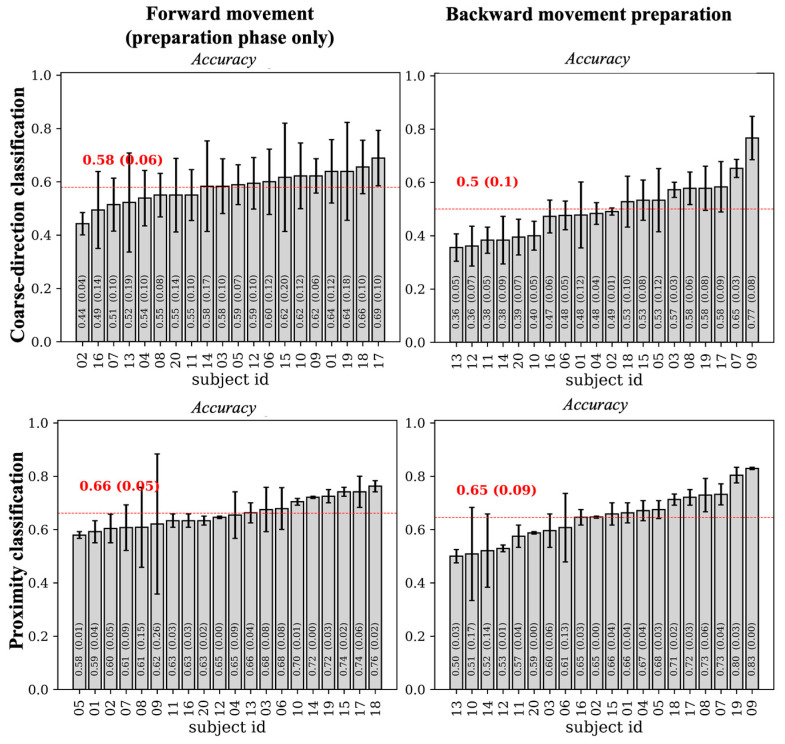
Neural network accuracy: forward vs. backward movement preparation. The left and right panels respectively show the decoding accuracy, while decoding the movement preparation in the forward and backward movements separately for the coarse-direction and proximity decoding problems. See the caption of Figure 4 for further details about the represented quantities.

**Figure 9:**
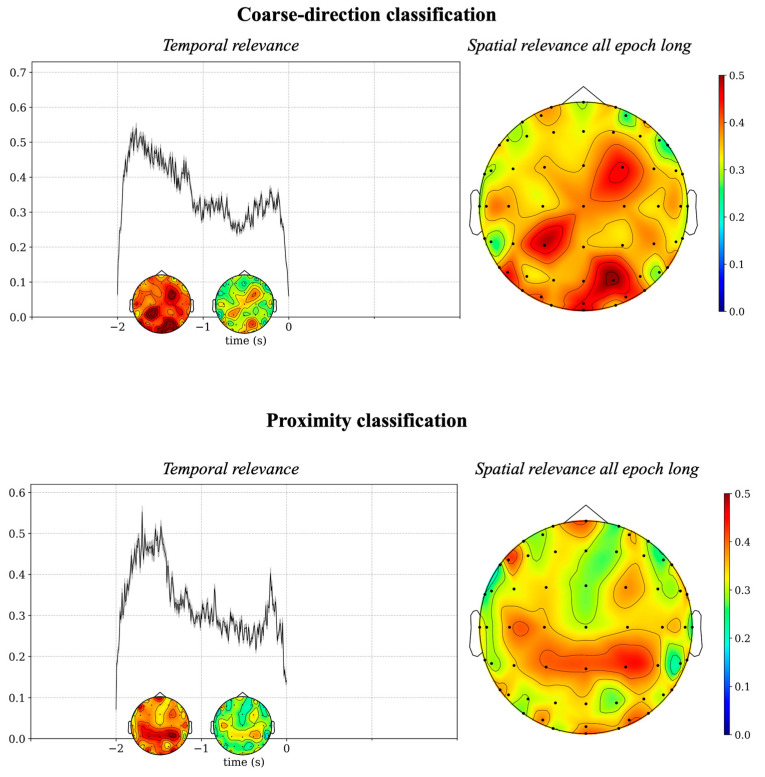
Explaining network decision in the backward movement preparation: temporal and spatial relevance. The relevance representations are reported separately for the coarse-direction and proximity decoding problems. See Figure 6 for details about the meaning of the representations.

Results of decoding accuracy for backward movement preparation epochs are shown in Figure 8 (right panels). The accuracy reached an average value across subjects of 0.5 and 0.65 in the coarse-direction classification problem and in the proximity classification problem, respectively. For each subject, the accuracy was above the chance level (0.33 and 0.5 in the two classification problems, respectively) in all subjects, except for one subject with accuracy at chance level. For a fair comparison, the left panels display the decoding accuracy values obtained for the forward movement epochs while occluding the motor execution phase, thus relative to the preparation phase. Under this matched condition, accuracy for the forward movement preparation was moderately above (0.58 in the three-class problem, *p* < 0.05) and comparable (0.66 in the two-class problem, *p* > 0.05) to that obtained for the backward movement preparation in the corresponding classification problem (0.5 and 0.65). The results suggest that the two preparation phases did not diverge markedly. This is also confirmed by the results of the explanation techniques. Figure 9 reports the results of DeepLift applied to the backward movement preparation separately in the coarse-direction classification problem and in the proximity classification problem. The relevance patterns obtained in case of the backward movement resembled those obtained in the forward movement preparation (Figure 6), being higher in the first second of the movement preparation phase than in the subsequent second and mainly involving parietal and central scalp regions.

## 4. Discussion

In this study, we investigated the potential of a CNN-based decoder to classify movement directions in a center-out reaching task, as well as the application of an explainability technique to uncover how directional information is encoded in the spatio-temporal features of EEG signals. The focus was on the forward movement. A preliminary ERSP analysis was employed to characterize the temporal evolution of alpha- and beta-band oscillations during the forward movement. Then, EEGNet, a widely adopted CNN for EEG decoding, was used to address different classification problems directly from the EEG time series: discrimination between five different reaching endpoints (fine-direction classification), three endpoints (coarse-direction classification), and two endpoints (proximity classification). The direction decoding was complemented by explanation techniques (DeepLIFT and occlusion tests) to quantify the spatial and temporal features of the EEG time series that contributed most to the decoding. The analyses conducted in this study, either the conventional ERSP analysis and CNN-based analysis, provide a comprehensive examination of center-out-reaching, encompassing both the motor planning and execution phases. To the best knowledge of the authors this is the first time that (i) a deep learning-based algorithm is used for fine direction decoding, testing its feasibility in multiple use-case scenarios, and (ii) an explainable deep learning framework is leveraged for investigating the directional encoding in EEG signals during center-out reaching, in a data-driven and end-to-end manner.

All the participant-level decoders developed in this study for forward movement decoding achieved classification performance well above the chance level (0.20, 0.33, and 0.50, respectively for the fine-direction, coarse-direction, and proximity classifications; see Figure 4), achieving average accuracies across participants of 0.45 (five classes), 0.64 (three classes) and 0.70 (two classes). These accuracy values deserve some comments. Indeed, although they were above the chance level, they still ended up inadequate for practically usable BCIs. In our study, decoding performance should be interpreted primarily as evidence of discriminative information contained in non-invasive EEG signals rather than as an indicator of immediate usability for multi-command BCI controls. Indeed, the primary aim of this study was to investigate EEG information discriminative of arm movement direction in order to provide results about direction-specific EEG signatures that may be prospectively useful for informing and guiding future non-invasive BCI studies, while a gap (especially in the case of the five-class decoding) remains between the current work performance and the level required for practical deployment. Substantially higher decoding accuracies for multi-class movement direction have often been reported in invasive BCI systems (using intracortical or electrocorticographic (ECoG) signals), due to intrinsic higher spatial resolution and better signal-to-noise ratio [39]. For example, Ball et al. [40] decoded movement direction from ECoG signals recorded while the subjects performed self-paced center-out reaching tasks, achieving an accuracy of 76% in the case of four-class decoding, and of about 60% in the case of eight-class decoding (accuracy became 45% and approximately 42% respectively when considering only the pre-movement period). In another ECoG study [41], decoding accuracy resulted in the range 56–83% for a four-class center-out reaching task. Moreover, in a reaching task towards eight targets (at the vertices of a 3D physical cube, with the starting position at the center), the final target position was predicted from ECoG signals with accuracy between 49% and 66.2% [42]. Finally, a very recent study [43] showed that direction of movements in a four-class center out reaching task could be predicted with 86% accuracy from human local field potentials during both movement planning and movement execution. These results evidence that invasive BCI approaches currently achieve decoding performances that are closer to practical usability for multi-class movement control, albeit at the cost of surgical invasiveness and associated clinical limitations. Our results, although settling at a lower level of performance compared to invasive approaches, support the presence of direction-specific discriminative information in non-invasive EEG signals, and reached decoding accuracy in line with previous studies on EEG-based direction decoding. For example, Úbeda et al. [14] obtained an accuracy of approximately 0.5 while classifying between four reaching endpoints (left vs. right vs. up vs. down). A similar value was found by Kobler et al. (accuracy of 0.56) [15] and Waldert et al. (accuracy of 0.55) [16] in the same classification problem. When addressing more challenging discrimination problems, involving more than four reaching endpoints to be classified, accuracies degraded down to 0.3 in Úbeda et al. [15] (eight reaching endpoints); in contrast, when addressing simpler decoding problems, accuracies increased between 0.65 in Li et al. [12] and 0.73 in Sagila et al. [13] (two reaching endpoints). Finally, it is worth noticing that in a four-endpoints direction decoding, higher classification accuracies can be achieved, up to 0.75, when using ultra-high-density EEG configuration (200 EEG channels), as reported in a recent study by Ma et al. [17]. This suggests that increasing electrode density can substantially improve class separability and decoding performance in non-invasive approaches, reaching value comparable to invasive approaches. In addition, integrating complementary sensing modalities (e.g., EEG and EMG) may improve accuracy, too. Thus, these represent potential strategies to progressively bridge the gap between current decoding performance in non-invasive approaches and future practical usability. While performance results in our study essentially match those of the previous non-invasive studies, most of them [12,14,16,17] presented the decoding results on a limited set participants (≤10) and are based on machine learning (with features selected a priori) rather than on deep learning (end-to-end) techniques. Therefore, the current study provides a depiction of center-out-reaching decoding on a wider sample. Moreover, our end-to-end approach that avoids a priori feature selection, combined with explainability techniques, enables the exploration of the relevance of all available EEG information, rather than confining the analysis to predefined features or assumptions.

Indeed, as an important point of novelty, besides the performance evaluation, this work also furnishes an interpretation of the decoding performance, by applying two different explanation techniques (DeepLIFT and occlusion tests).

Concerning the time domain, the DeepLIFT-based relevance representations (see Figure 5 and Figure 6) suggest that the most relevant time interval was in the motor preparation phase, approximately from −2 s to −1 s, corresponding to the first second after the direction cue. Notably, an additional interval of high relevance appeared in the execution phase, approximately from 0 s to 1 s—that is, the first second immediately after the go signal, although the relevance in this interval was lower than in the preparation phase. The greater relevance of the preparatory phase is confirmed by the occlusion tests (see Figure 7), showing a larger accuracy reduction when removing the EEG signals in the preparation phase than in the execution phase, up to −0.13 (remotion of preparatory phase) vs. −0.06 (remotion of execution phase) across classification problems. Concerning the spatial domain, the DeepLIFT-based relevance representations (see Figure 5 and Figure 6) highlighted the parietal and parietal–occipital EEG channels as the most relevant ones, especially in the time interval showing the highest relevance (from −2 s to −1 s).

These spatio-temporal insights obtained from the CNN coupled with explanation techniques parallel the results from the ERSP analysis (see Figure 3). In particular, according to ERSP analysis, in the preparation phase (from −2 s to 0 s) the scalp regions exhibiting significant difference among the reaching endpoints were predominantly posterior at the parietal–occipital electrode sites, not only in the alpha band but also in the beta band (see violet dots in Figure 3). Moreover, these differences were confined to the first part (from −2 s up to −0.5 s) of the preparation phase. In contrast, in the first second after the go signal (from 0 s to 1 s), the differences were more confined to the beta-band (and located centrally), but without surviving the correction for multiple tests. Overall, two considerations can be derived. First, although central beta-band modulations were present (both during preparation and execution), they were less robust than posterior modulations. Second, in line with the results of CNN-based analysis, ERSP analysis suggests that direction-related modulatory processes during movement execution were less robust than the direction-related preparatory process. In addition to this interesting match between the CNN-based and ERSP-based EEG analyses, our results are also in line with the findings of prior studies. Indeed, a stronger representation of movement direction was found during movement preparation than movement initiation/execution, 200–300 ms after direction in Wang and Makeig [44] or 300–400 ms after the direction cue in Kobler et al. [15]. Moreover, the same studies found that the parietal–occipital areas were encoding most of the directional information, and that this encoding was stronger than in somatosensory areas. In fact, the posterior parietal cortex in humans and non-human primates hosts areas involved in the visuomotor processes required to generate action plans [45,46,47].

It is worth noticing that we conducted a supplementary analysis on the backward movement to disentangle the contribution of visual process and motor intention in decoding the movement preparation phase. In particular, our results suggest that although visually evoked information may have partially contributed to decoding performance during forward movement preparation, a substantial portion of the discriminative information appeared to be related to motor intention. This can be inferred from the substantial matching between the results of forward movement preparation and backward movement preparation (not contaminated by visually evoked information), both in terms of decoding performance (Figure 8) and relevance representation (Figure 6 and Figure 9).

Overall, this study highlights that EEG signals contain discriminative information about forward movement direction, that can be adequately captured and decoded by CNNs. Moreover, our data-driven, end-to-end approach advances the understanding of the EEG correlates of center-out reaching. This could be highly valuable for (i) advancing knowledge on the neurophysiology of reaching movements; (ii) providing indications on the most informative EEG channels and time intervals to target in order to maximize discriminatory power (i.e., decoding accuracy) for EEG-based direction decoding during reaching tasks. Indeed, as concerning the last point, our findings could provide guidance to neuroscientists for the design of novel decoding pipelines by enabling more accurate and targeted feature extraction. This knowledge could be exploited to optimize decoding strategies, thereby supporting, prospectively, the development of more naturalistic, robust, and high-performance BCIs.

Although the results obtained are promising, a number of aspects not considered in the present study remain to be addressed and may represent interesting directions for future investigations.

Firstly, we did not control for factors, such as subject-specific physiological or neurophysiological characteristics, that may increase inter-subject heterogeneity, thus possibly limiting the consistency of the observed effects across participants. For example, the hormonal status of the female participants (nine out of twenty participants), which may influence cortical excitability and spectral EEG characteristics [48], was not taken into account, and we did not evaluate individual alpha and beta frequency ranges based on individualized alpha frequency peaks. These factors may have contributed to inter-individual variability in EEG features and, consequently, to differences in decoding performance from one subject to another (up to about 30% difference; see bar-plots in Figure 4). Furthermore, the DeepLIFT analysis was applied across all participants in order to identify spatial and temporal EEG features that were consistently relevant for classification at the entire group level. As a result, inter-subject heterogeneity may have reduced the relevance of some features. Future studies may benefit from explicitly accounting for subject-specific factors that can influence EEG signal characteristics, in order to reduce inter-subject variability and possibly promoting more stable decoding performance and more consistent group-level relevance patterns.

Another important aspect concerns different characteristics and roles of alpha sub-bands in movement—namely low and high alpha bands (below and above the alpha peak frequency). Previous studies have reported different response patterns of low and high alpha band activity both in movement execution and observation, suggesting that alpha band activity during movement is not a unitary phenomenon but rather that different alpha sub-bands are functionally dissociated and reflect different contributions [49,50,51,52]. While in this study we did not examine different alpha sub-bands, in future works it may be of high interest to investigate their distinct contributions to movement-related EEG decoding. To this aim, it would be advantageous to use neural network decoders designed to be directly interpretable also in the frequency domain (see for example our recent studies [53,54]). In this way, it would be possible to disentangle the relative relevance of low- and high-alpha sub-bands for decoding reaching movements, both during movement preparation and execution. This could be relevant not only for advancing the neurophysiological understanding of the functional role of alpha sub-bands in motor control, but also it could have prospectively practical implications in the context of neurorehabilitation. Of course, for the analysis of alpha sub-bands, an essential step would be the subject-specific estimation of the individual alpha peak frequency, for subject-specific identification of the low- and high-alpha sub-bands.

Another aspect that deserves comment is the possible contamination of EEG recordings by EMG activity during movement execution, particularly in the beta frequency range. Indeed, previous studies have shown significant cortico-muscular coupling during voluntary movement and muscular contraction, especially in the beta but also in the gamma band, reflecting functional communication between cortical and peripheral activity [55,56,57]. Importantly, methods that examine direction of information flow evidence a bidirectional influence, both from EEG to EMG and from EMG to EEG. In the present study, ICA has been applied as a standard preprocessing step to minimize non-neural artifacts (including EMG artifacts). However, cortico-muscular coupling or mixed neural–peripheral activity, which still may present characteristics of neural origin, are likely preserved in the data. From a decoding perspective, such mixed EEG–EMG signals may still carry behaviorally relevant information for movement discrimination. However, this factor should be taken into account when interpreting the neurophysiological origin of the features contributing to classification performance, as EEG-based decoding may rely not only on purely cortical activity but also on peripheral contributions mixed within the EEG signal. An interesting direction for future work would be to consider EEG-based decoding together with measures of cortico-muscular coupling within the same experimental paradigm, to investigate complementary contributions of cortical and peripheral components to movement discrimination.

Finally, in this study, we used a within-subject training strategy. This strategy was adopted to isolate task-related information while mitigating the strong inter-subject variability typical of EEG data. This approach allowed the CNN to better accommodate individual neurophysiological patterns, and is the prevailing paradigm in non-invasive BCI research and applications, where decoding models are typically trained and calibrated on data from the same subject. On the contrary, cross-subject training as well as transfer learning (which exploits models pretrained on other individuals and fine-tuned on a new one) were not addressed in the present work, although they may become relevant when generalization across users is required, e.g., in zero- or reduced-calibration settings. The high inter-subject variability observed in our results (up to 30% differences in decoding accuracy across subjects) suggests that cross-subject generalization represents a particularly challenging aspect of this decoding problem, which can be addressed in future studies.

## 5. Conclusions

In this work, we demonstrated the feasibility of decoding directional information in center-out reaching from EEG recordings using a CNN. We addressed multiple decoding scenarios of increasing complexity, ranging from proximity-based discrimination (two classes) to fine direction decoding (five classes). Across all scenarios, the proposed approach achieved classification accuracies consistently above chance level, confirming that meaningful directional information can be extracted from EEG signals even in challenging settings. Beyond decoding performance, a key contribution of this study lies in the integration of explainability techniques to investigate the EEG correlates of center-out reaching. Our data-driven approach combined with explanation analyses revealed that directional information is predominantly encoded during the movement preparation phase, with a secondary contribution during early execution. Spatially, the most informative features were mainly localized over parietal and parietal–occipital regions, in agreement with the results obtained from conventional ERSP analysis and with prior evidence on directional encoding in center-out reaching.

Overall, these findings advance the understanding of how reaching direction is represented in EEG signals and highlight the potential of explainable deep learning models as tools for both decoding and neuroscientific investigation. The insights provided here may inform the design of more targeted feature extraction strategies and contribute to the development of more accurate and interpretable EEG-based BCIs for naturalistic motor control.

## Figures and Tables

**Table 1:** EEGNet architecture: layer details. The table reports the name, main hyper-parameters, and output shape for each layer. Unless specified, unit stride and padding are applied. ‘Activation = linear’ denotes linear activation function of the neurons. Note that ‘n. classes’ varies according to the classification problem.

Block ID	Layer Name	Main Hyper-Parameters	Output Shape
	*Input*	-	(1, 60, 5 s × 128 Hz = 640)
1	*Time-Conv2D*	n. filters = 8; filter length = 16; activation = linear	(8, 60, 640)
	*BatchNorm2D*	-	(8, 60, 640)
	*Space-DepthConv2D*	n. filters = 16; filter length = 60; activation = linear	(16, 1, 640)
	*BatchNorm2D*	-	(16, 1, 640)
	*ELU*	-	(16, 1, 640)
	*Time-AvgPool2D*	filter length = 4	(16, 1, 160)
	*Dropout*	dropout rate = 0.1	(16, 1, 160)
2	*Time-SepConv2D*	n. filters = 16; filter length = 8; activation = linear	(16, 1, 160)
	*BatchNorm2D*		(16, 1, 160)
	*ELU*		(16, 1, 160)
	*Time-AvgPool2D*	filter length = 4	(16, 1, 40)
	*Dropout*	dropout rate = 0.1	(16, 1, 40)
	*Flatten*		640
3	*Fully-connected*	n. classes = 2, 3, 5	2, 3, 5
	*Softmax*	-	2, 3, 5

**Table 2:** Neural network performance metrics. The accuracy, F1-score, and AUC are reported separately for each subject and each classification problem (each value represents the mean across folds). Bold values in the last row indicate the mean across participants and standard deviation in parentheses.

Subject ID	Fine Direction Classification			Coarse Direction Classification			Proximity Classification		
	*Accuracy*	*F*1*-Score*	*AUC*	*Accuracy*	*F*1*-Score*	*AUC*	*Accuracy*	*F*1*-Score*	*AUC*
1	0.43	0.57	0.75	0.73	0.83	0.87	0.64	0.77	0.83
2	0.40	0.55	0.71	0.53	0.68	0.69	0.64	0.77	0.84
3	0.50	0.65	0.82	0.68	0.80	0.84	0.70	0.82	0.88
4	0.37	0.51	0.72	0.53	0.67	0.72	0.60	0.75	0.81
5	0.40	0.54	0.74	0.66	0.78	0.83	0.70	0.82	0.89
6	0.42	0.56	0.74	0.59	0.72	0.78	0.66	0.80	0.86
7	0.41	0.56	0.70	0.56	0.71	0.75	0.68	0.80	0.87
8	0.52	0.66	0.83	0.68	0.80	0.86	0.67	0.80	0.86
9	0.41	0.56	0.72	0.63	0.76	0.79	0.70	0.82	0.88
10	0.46	0.60	0.76	0.68	0.79	0.84	0.76	0.86	0.92
11	0.38	0.53	0.69	0.56	0.70	0.72	0.64	0.77	0.83
12	0.45	0.61	0.76	0.60	0.73	0.79	0.67	0.79	0.85
13	0.45	0.61	0.75	0.58	0.72	0.76	0.75	0.85	0.91
14	0.42	0.57	0.76	0.62	0.75	0.79	0.73	0.84	0.90
15	0.48	0.62	0.80	0.81	0.89	0.95	0.79	0.88	0.94
16	0.37	0.52	0.68	0.60	0.74	0.82	0.67	0.80	0.86
17	0.63	0.76	0.89	0.77	0.86	0.93	0.80	0.87	0.93
18	0.50	0.65	0.79	0.73	0.83	0.90	0.74	0.85	0.91
19	0.56	0.70	0.87	0.73	0.84	0.91	0.76	0.86	0.91
20	0.36	0.52	0.69	0.52	0.66	0.76	0.68	0.81	0.87
	**0.45 (0.07)**	**0.59 (0.06)**	**0.76 (0.06)**	**0.64 (0.08)**	**0.76 (0.06)**	**0.82 (0.07)**	**0.70 (0.05)**	**0.82 (0.04)**	**0.88 (0.04)**

## Data Availability

The data presented in this study are available on request from the corresponding author.
